# Supplementary material for: Proteomes of Residual Tumors in Curcumin-Treated Rats Reveal Changes in Microenvironment/Malignant Cell Crosstalk in a Highly Invasive Model of Mesothelioma
Source: Int J Mol Sci. 2022 Nov 8;23(22):13732. doi: 10.3390/ijms232213732 (PMC9691155; doi:10.3390/ijms232213732)
Supplement: Supplementary file 1 [file ijms-23-13732-s001.zip › ijms-1959668-supplementary.pdf]

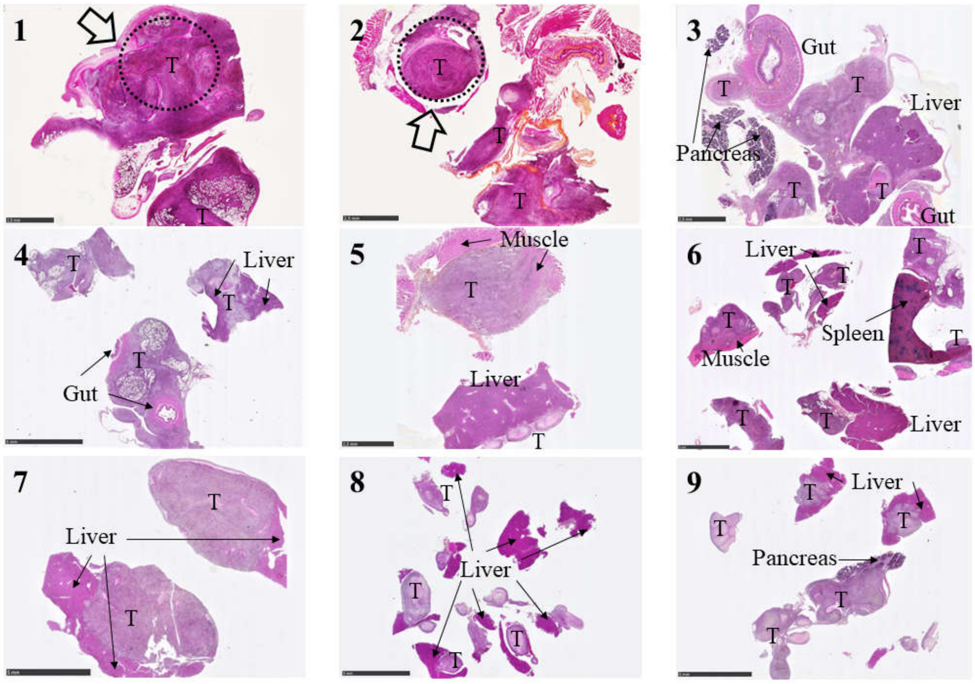

Figure S1. General views of M5-T1 tumors from the group of untreated rats (HPS staining, scale bars at the bottom left corner of each photograph). Samples **1** and **2** were selected for the study according to criteria described in sections 4.2 and 4.3 (Materials and methods). The dashed black circles (arrows) represent areas of interest, used both for histological (morphology of tumor infiltrating lymphocytes) and proteomic analyses. T = tumor.

Table S1.

Supplementary Table S1: Complete list of proteins exhibiting common abundance changes under curcumin treatment. ↑ and ↓ symbolize significant ($p < 0.05$) common increase or decrease relative to tumors from untreated rats.

Gene	Full name	G2/G1, G3/G1
Impdh1	Inosine-5′-monophosphate dehydrogenase 1	↑
Pld3	5′-3′ exonuclease PLD3	↑
F3	Tissue factor	↑
Rbp4	Retinol-binding protein 4	↑
Tf	Serotransferin	↑
Cav1	Caveolin-1	↑
Ankh	Progressive ankylosis protein homolog	↑
Cacna2d1	Voltage-dependent calcium channel subunit alpha-2/delta-1	↑
Cpne1	Copine-1	↑
Tmem231	Transmembrane protein 231	↑
Ctsl	Procathepsin L	↑
Rasgrf1	Ras-specific guanine nucleotide-releasing factor 1	↑
Rraga	Ras-related GTP-binding protein A	↑

Fkbp6	Inactive peptidyl-prolyl cis-trans isomerase FKBP6	↑
Mmgt1	ER membrane protein complex subunit 5	↑
Arl6ip6	ADP-ribosylation factor-like protein 6-interacting protein 6	↑
Dab2	Disabled homolog 2	↑
Map2k2	Dual specificity mitogen-activated protein kinase kinase 2	↑
Pip4k2c	Phosphatidylinositol 5-phosphate 4-kinase type-2 gamma	↑
Cdh2	Cadherin-2	↑
Epdr1	Mammalian ependymin-related protein 1	↑
Nptn	Neuroplastin	↑
Emd	Emerin	↑
Aprt	Adenine phosphoribosyltransferase	↑
Akr1b7	Aldo-keto reductase family 1 member B7	↑
Dhcr24	Delta(24)-sterol reductase	↑
Enpp1	Ectonucleotide pyrophosphatase/phosphodiesterase family member 1	↑
Spr	Sepiapterin reductase	↑
Slc27a1	Long-chain fatty acid transport protein 1	↑
Acadvl	Very long-chain specific acyl-CoA dehydrogenase, mitochondrial	↑
Ech1	Delta(3,5)-Delta(2,4)-dienoyl-CoA isomerase, mitochondrial	↑
D2hgdh	D-2-hydroxyglutarate dehydrogenase, mitochondrial	↑
Gpd2	Glycerol-3-phosphate dehydrogenase, mitochondrial	↑
Ndufs1	NADH-ubiquinone oxidoreductase 75 kDa subunits, mitochondrial	↑
Pdk1	[Pyruvate dehydrogenase (acetyl-transferring)] kinase isoenzyme 1, mitochondrial	↑
Plgrkt	Plasminogen receptor	↑
Prdx5	Peroxiredoxin-5, mitochondrial	↑
Sardh	Sarcosine dehydrogenase, mitochondrial	↑
Mrppl23	39S ribosomal protein L23, mitochondrial	↑
Mrps25	28S ribosomal protein S25, mitochondrial	↑
Igf2bp1	Insulin-like growth factor 2 mRNA-binding protein 1	↑
Lsg1	Large subunit GTPase 1 homolog	↑
Rpl13a	60S ribosomal protein L13a	↑
Rpl18	60S ribosomal protein L18	↑
Rpl19	60S ribosomal protein L19	↑
Rps4x	40S ribosomal protein S4, X isoform	↑
Mphosph8	M-phase phosphoprotein 8	↑
Psmb5	Proteasome subunit beta type-5	↑
Psmb6	Proteasome subunit beta type-6	↑
Flnc	Filamin-C	↑
Apaf1	Apoptotic protease-activating factor 1	↓
Casp1	Caspase-1	↓
Cd74	H-2 class II histocompatibility antigen gamma chain	↓
Erap1	Endoplasmic reticulum aminopeptidase 1	↓
Fcer1g	High affinity immunoglobulin epsilon receptor subunit gamma	↓
Fcgr3a	Low affinity immunoglobulin gamma Fc region receptor subunit gamma	↓
Fcn2	Ficolin-2	↓
Gbp2	Guanylate-binding protein 1	↓
Gimap4	GTPase IMAP family member 4	↓
Irgm	Immunity-related GTPase family M protein	↓
Nampt	N-acylethanolamine-hydrolyzing acidamidase	↓

Rassf2	Ras association domain-containing protein 2	↓
Rgs19	Regulator of G-protein signaling 19	↓
RT1-Aw2	Class I histocompatibility antigen, Non-RT1.A alpha-1 chain	↓
RT1-Bb	Rano class II histocompatibility antigen, B-1 beta chain	↓
RT1-Db1	Rano class II histocompatibility antigen, D-1 beta chain	↓
Serpinb1a	Leukocyte elastase inhibitor A	↓
Sh3bp1	SH3 domain-containing protein 1	↓
Sting1	Stimulator of interferon genes protein	↓
Stk17b	Serine/threonine-protein kinase 17B	↓
Tap1	Antigen peptide transporter 1	↓
Tap2	Antigen peptide transporter 2	↓
Trafd1	TRAF-type zinc finger domain-containing protein 1	↓
Vcam1	Vascular cell adhesion protein 1	↓
Wars1	Tryptophan t-RNA ligase, cytoplasmic	↓
A1bg	Alpha-1B-glycoprotein	↓
B2m	Beta-2-microglobulin	↓
Hp	Haptoglobin	↓
Lifr	Leukemia inhibitory factor receptor	↓
Rab27a	Ras-related protein Rab-27A	↓
Bst2	Bone marrow stromal antigen 2	↓
Pak1	Serine/threonine-protein kinase PAK1	↓
Mcfd2	Multiple coagulation factor deficiency protein 2 homolog	↓
Ssr3	Translocon-associated protein subunit gamma	↓
GLTP	Glycolipid transfer protein	↓
Mob4	MOB-like protein phocein	↓
Rbp1	Retinol-binding protein 1	↓
Lap3	Cytosol aminopeptidase	↓
Dock9	Dedicator of cytokinesis protein 9	↓
Ltbp1	Latent-transforming growth factor beta-binding protein 1	↓
Wdr91	WD repeat-containing protein 91	↓
Numb	Protein numb homolog	↓
Hagh	Hydroxyacyl glutathione hydrolase, mitochondrial	↓
Naaa	N-acylethanolamine-hydrolyzing acidamidase	↓
Pgd	6-phosphogluconate dehydrogenase, decarboxylating	↓
Rnpep	Aminopeptidase B	↓
Uggt1	UDP-glucose: glycoprotein glucosyltransferase 1	↓
Xdh	Xanthine dehydrogenase/oxidase	↓
Acsf2	Medium-chain acyl-CoA ligase ACSF2, mitochondrial	↓
Bdh1	D-beta-hydroxybutyrate dehydrogenase, mitochondrial	↓
Mfn1	Mitofusin-1	↓
Nmes1	Normal mucosa of esophagus-specific gene 1 protein	↓
Celf2	CUGBP Elav-like family member 2	↓
Dnajc8	Dnaj homolog subfamily C member 8	↓
Iah1	Isoamyl acetate-hydrolyzing esterase 1 homolog	↓
Ptbp3	Polypyrimidine tract-binding protein 3	↓
Sub1	Activated RNA polymerase II transcriptional coactivator p15	↓
Psmb10	Proteasome subunit beta type-10	↓
Psme1	Proteasome activator complex subunit 1	↓
Psme2	Proteasome activator complex subunit 2	↓
Add3	Gamma-adducin	↓

Cotl1	Coactosin-like protein	↓
Entpd1	Ectonucleoside triphosphate diphosphohydrolase I	↓
Evl	Ena/VASP-like protein	↓
Fnbp1	Formin-binding protein 1	↓
Mapre2	Microtubule-associated protein RP/EB family member 2	↓

Table S2.

Supplementary Table S2: Complete list of proteins exhibiting specific abundance changes in G3 rats (multiple early curcumin treatment). ↑ and ↓ symbolize significant common significant ($p < 0.05$) increase or decrease in G3 vs G1 (untreated rats) and G3 vs G2 (rats receiving two late curcumin injections), but not in G2 vs G1.

**Gene**	**Full name**	Change
Cd276	CD276 antigen	↑
Il33	Interleukin-33	↑
Mif	Macrophage migration inhibitory factor	↑
Lrp1	Prolow-density lipoprotein receptor-related protein 1	↑
Lrrfip1	Leucine-rich repeat flightless-interacting protein 1	↑
Ptms	Parathymosin	↑
Col5a1	Collagen alpha-1(V) chain	↑
Gpc1	Glypican-1	↑
Mrc2	C-type mannose receptor 2	↑
Ecm1	Extracellular matrix protein 1	↑
Gtf2i	General transcription factor II-I	↑
Hrg	Histidine-rich glycoprotein	↑
Ptpn11	Tyrosine-protein phosphatase non-receptor type 11	↑
Mapk3	Mitogen-activated protein kinase 3	↑
Grip1	Glutamate receptor-interacting protein 1	↑
Mxra8	Matrix remodeling-associated protein 8	↑
Adcy3	Adenylate cyclase type 3	↑
Pdcl	Phosducin-like protein	↑
Rin1	Ras and Rab interactor 1	↑
S100a10	Protein S100-A10	↑
Pdlim3	PDZ and LIM domain protein 3	↑
Fabp5	Fatty acid-binding protein 5	↑
Cavin1	Caveolae-associated protein 1	↑
Map1lc3b	Microtubule-associated proteins 1A/1B light chain 3B	↑
Os9	Protein OS-9	↑
Adamtsl4	ADAMTS-like protein 4	↑
Aga	N(4)-beta-N-acetylglucosaminyl)-L-asparaginase	↑
Oga	Protein O-GlcNAcase	↑
Gpi	Glucose-6-phosphate isomerase	↑
Pgm1	Phosphoglucomutase-1	↑
Irf2bpl	Interferon regulatory factor 2 binding protein like	↑
Niban2	Protein Niban 2	↑
Ahr	Aryl hydrocarbon receptor	↑

Abhd14b	Putative protein-lysine deacylase ABHD14B	↑
Psmc3ip	Homologous-pairing protein 2 homolog	↑
Nab1	NGFI-A-binding protein 1	↑
Sorbs2	Sorbin and SH3 domain-containing protein 2	↑
Synpo2	Synaptopodin-2	↑
Dnm3	Dynamin-3	↑
Gsn	Gelsolin	↑
Dpysl3	Dihydropyrimidinase-related protein 3	↑
Phldb1	Pleckstrin homology-like domain family B member 1	↑
Pcmt1	Protein-L-isoaspartate(D-aspartate) O-methyltransferase	↑
Dnm1	Dynamin-1	↑
Jup	Junction plakoglobin	↑
Cd47	Leukocyte surface antigen CD47	↓
Il18	Interleukin-18	↓
Il1rn	Interleukin-1 receptor antagonist protein	↓
Coro1a	Coronin-1A	↓
Lyz1	Lysozyme C-1	↓
Icam1	Intercellular adhesion molecule 1	↓
Ptprc	Receptor-type tyrosine-protein phosphatase C	↓
Ifitm3	Interferon-induced transmembrane protein 3	↓
Arrb2	Beta-arrestin-2	↓
Gnai2	Guanine nucleotide-binding protein G(i) subunit alpha-2	↓
Hk3	Hexokinase-3	↓
Tmed5	Transmembrane emp24 domain-containing protein 5	↓
Gclm	Glutamate—cysteine ligase regulatory subunit	↓
Atp5mg	ATP synthase subunit g, mitochondrial	↓
Atp5pf	ATP synthase-coupling factor 6, mitochondrial	↓
Cox7a2	Cytochrome c oxidase subunit 7A2, mitochondrial	↓
Idh2	Isocitrate dehydrogenase [NADP], mitochondrial	↓
Sdhd	Succinate dehydrogenase [ubiquinone] cytochrome b small subunit, mitochondrial	↓
Thy1	Thy-1 membrane glycoprotein	↓
Stat3	Signal transducer and activator of transcription 3	↓
